# Fibrinogen and catheter advancement difficulty: unveiling the key predictors of early *in-situ* bleeding after peripherally inserted central catheter insertion

**DOI:** 10.3389/fmed.2026.1836789

**Published:** 2026-07-15

**Authors:** Shusheng Jiao, Hongjie Yang, Jianling Yang, Jianxia Zhi

**Affiliations:** 1 Department of Neurology, Bethune International Peace Hospital, Shijiazhuang, Hebei, China; 2 Department of Medical Oncology, Bethune International Peace Hospital, Shijiazhuang, Hebei, China; 3 Department of General Surgery, Bethune International Peace Hospital, Shijiazhuang, Hebei, China

**Keywords:** catheter advancement, fibrinogen, *in-situ* hemorrhage, peripherally inserted central catheter (PICC), tumor

## Abstract

**Background:**

Peripherally inserted central catheter (PICC) is widely used for medium-to-long-term venous access, but early puncture site bleeding within 48 h remains a common complication, potentially leading to increased morbidity and healthcare costs. Identifying independent risk factors is crucial for developing targeted prevention strategies.

**Methods:**

In this single-center, retrospective study, a consecutive cohort of patients who successfully underwent PICC insertion between March 2024 and March 2025 was enrolled. The study population was divided based on the criterion of *in-situ* hemorrhage into two cohorts for comparison: one with *in-situ* hemorrhage within 48 h post-insertion, and one without. Patient-related (demographics, comorbidities, laboratory parameters, medication history) and procedure-related factors (punctured vein, number of attempts, catheter dislodgement, etc.) were collected. Univariate analyses identified significant variables, which were subsequently entered into multivariable logistic regression models to identify independent predictors.

**Results:**

A total of 375 patients were included in the final analysis, with an *in-situ* hemorrhage incidence of 21.9%. Multivariable analysis confirmed lower fibrinogen level as an independent risk factor (OR = 0.762 per 1 g/L increase, 95% CI: 0.583–0.995, *p* = 0.046) and catheter advancement difficulty as a significant procedural predictor (OR = 2.560, 95% CI: 1.010–6.485, *p* = 0.047). An unexpected association between tumor diagnosis and reduced bleeding risk was also observed (OR = 0.331, 95% CI: 0.142–0.768, *p* = 0.011); given its counterintuitive nature and the limitations of the study design, this finding should be regarded as hypothesis-generating rather than conclusive. The number of puncture attempts showed a trend toward significance (OR = 1.661, *p* = 0.060).

**Conclusion:**

Lower fibrinogen level and catheter advancement difficulty are independent risk factors for early PICC-related bleeding, while a tumor diagnosis appears protective. These findings highlight the importance of pre-procedural coagulation assessment and meticulous catheter placement.

## 1 Introduction

Peripherally inserted central catheter (PICC) has emerged as a cornerstone of modern clinical practice, providing a safe, reliable, and cost-effective medium-to-long-term venous access for patients requiring vesicant infusion, prolonged chemotherapy, parenteral nutrition, or repeated blood transfusions (1). By inserting the catheter tip into the lower 1/3 of the superior vena cava or its junction with the right atrium via peripheral veins such as the basilic, median cubital, or cephalic veins, PICC minimizes peripheral vascular damage from repeated punctures and ensures efficient delivery of therapeutic agents (2). The advantage of PICC also stems from a relatively low complication rate and high cost-effectiveness compared to other central venous access devices (3). This technique, classified as a low-risk Level 1 procedure (4), has been widely adopted in modern healthcare delivery across both inpatient and outpatient settings, with mature operational protocols and professional vascular access teams facilitating its application.

Despite their overall safety profile, PICC placement is an invasive procedure not without risks. Common complications include catheter-related bloodstream infection (5), venous thrombosis (6–8), and mechanical issues such as occlusion or malposition (9). Among the early and most frequently encountered complications is puncture site bleeding within the first 48 h post-insertion (10). While often self-limiting, post-procedural bleeding can range from minor oozing to significant hematoma formation. Clinically significant bleeding poses a substantial concern as it can lead to catheter dislodgement, necessitate dressing changes that increase infection risk, cause patient anxiety and discomfort, and in severe cases, result in premature catheter removal, thereby delaying critical therapy and increasing healthcare costs (11). The effective prevention and management of early bleeding are therefore crucial for optimizing PICC-related outcomes.

Existing literature has preliminarily linked puncture site bleeding to multiple factors, encompassing patient-related conditions, pharmacotherapeutic factors, procedural techniques, and post-insertion care (11). Patient factors such as thrombocytopenia, coagulation dysfunction, liver impairment, and malnutrition have been consistently cited as key contributors, as they impair the body’s hemostatic capacity (12). Procedural elements, including the selection of insertion site (e.g., avoiding the elbow fossa to reduce motion-induced vascular trauma), puncture technique, and the number of venipuncture attempts, also significantly influence bleeding risk (10). Postoperatively, inadequate compression time, inappropriate bandaging tension, and premature limb movement further exacerbate bleeding potential. Furthermore, the widespread use of therapeutic anticoagulants (e.g., warfarin, direct oral anticoagulants) and antiplatelet agents (e.g., clopidogrel, aspirin) in patients with cardiovascular or ischemic stroke presents a significant clinical challenge, as these medications directly increase the risk of bleeding complications (13).

However, the existing evidence is not entirely conclusive. Many studies investigating PICC complications often amalgamate bleeding with other events or focus on a limited set of variables, leaving a gap in a comprehensive, multivariate analysis dedicated specifically to early bleeding. Therefore, the primary objective of this single-center, retrospective study is to comprehensively identify and analyze the factors associated with puncture site bleeding within 48 h of PICC insertion, and to generate scientific evidence that can inform the development of targeted preventive strategies and evidence-based nursing protocols.

## 2 Methods and materials

### 2.1 Study design

This was a retrospective observational clinical study conducted at a single center in China, with a study period of 12 months. The study protocol was reviewed and approved by the Ethics Committee of our hospital (2023-KY-121); given the retrospective nature of the study (which involved reviewing and analyzing medical records of patients admitted to the hospital during the specified timeframe), the requirement for obtaining written informed consent from participants or their legal representatives was waived. During the study period, the data of eligible patients were collected and analyzed to achieve the preset research objectives.

A consecutive series of patients who underwent successful PICC placement and completed follow-up at the PICC clinic of our hospital between March 2024 and March 2025 were retrospectively enrolled and included in the final analysis based on the inclusion and exclusion criteria. Early *in-situ* hemorrhage was defined as any visible active bleeding or oozing from the puncture site requiring additional gauze packing or premature dressing change, or the formation of a new subcutaneous ecchymosis/hematoma (>2 cm in diameter) observed during the routine 48-h post-insertion dressing inspection. The study population was divided based on the above criterion into two cohorts for comparison: one with *in-situ* hemorrhage within 48 h post-insertion, and one without.

The patients (≥14 years) who met the indications for PICC placement and successfully underwent the procedure were included. Eligible participants were those: (1) undergoing their first-ever PICC insertion; (2) catheterized by senior PICC team operators (each with experience of ≥30 previous insertions) and receiving specialized nursing care; (3) implanted with a unified single-lumen 4-French catheter from the same manufacturer; (4) undergoing ultrasound-guided insertion using the modified Seldinger technique; (5) receiving manual compression for 10 min post-procedure; and (6) having complete bleeding observation records within 48 h after insertion.

Patients were excluded if they: (1) had known hereditary bleeding disorders (e.g., hemophilia); (2) received systemic hemostatic agents within 24 h prior to PICC insertion; (3) presented with skin breakdown or infection at the puncture site; (4) exhibited skin ecchymosis at or beyond the designated puncture area pre-procedure; (5) experienced other severe PICC-related complications (e.g., bloodstream infection, venous thrombosis, vascular perforation); (6) missed any key laboratory data (including liver function, renal function, coagulation profile, and platelet indices) drawn within the 3-day window before the procedure; or (7) had a history of PICC placement at our institution or another facility.

### 2.2 Collection of patent-related data

Patient-related factors were collected, encompassing demographic characteristics (age and sex), primary diagnoses, relevant comorbidities, laboratory parameters, and medication history. Primary diagnoses included malignancies, acute cerebral infarction, intracranial infections, and other diseases. Relevant comorbidities specifically comprised hypertension, diabetes mellitus, coronary heart disease, and hyperlipidemia. For laboratory parameters, liver function indicators (alanine aminotransferase, AST; aspartate aminotransferase, ALT; and albumin), renal function markers (creatinine and blood urea nitrogen), coagulation profile indices (prothrombin time, PT; activated partial thromboplastin time, APTT; thrombin time, TT; fibrinogen; D-dimer; and prothrombin activity, PTA), and platelet indices (platelet count, plateletcrit, PCT; mean platelet volume, MPV; platelet distribution width, PDW; and platelet-large cell ratio, P-LCR) were all recorded. Medication history documentation covered the use of anticoagulants (warfarin, heparin, and direct oral anticoagulants, DOACs) and antiplatelet agents (e.g., aspirin, clopidogrel, cilostazol, ticagrelor, indobufen).

### 2.3 Collection of procedure-related data

We also collected procedure-related data for all enrolled patients. This included the specific vein punctured (basilic, cephalic, median cubital, or brachial vein), the number of puncture attempts, occurrences of catheter displacement and difficult catheter advancement, and the daily frequency of infusions. Additionally, muscle tone in the limb used for PICC insertion was documented and classified as normal, decreased, or increased. Difficult catheter advancement was defined as ≥3 advancement attempts or documented resistance requiring withdrawal.

### 2.4 Statistical methods

All statistical analyses were conducted using SPSS version 24.0 (IBM Corp., Armonk, NY, USA). A two-sided *p*-value of less than 0.05 was considered statistically significant. Continuous variable normality was assessed via Kolmogorov-Smirnov test; descriptive statistics presented normally-distributed data as mean ± standard deviation (SD), non-normal as median (IQR), and categorical data as frequencies (%). Univariate analysis was performed to compare between the two groups. Continuous variables were analyzed using the Student’s *t*-test or the Mann-Whitney U test, as appropriate. Categorical variables were compared using the χ^2^ test or Fisher’s exact test. Candidate variables for multivariable analysis were selected based on clinical relevance derived from prior evidence and biological plausibility, supplemented by univariate screening (*p* < 0.1). This approach aimed to balance statistical efficiency with the inclusion of clinically meaningful covariates. The selected variables were subsequently entered into a multivariate logistic regression model to identify independent risk factors for *in-situ* hemorrhage. The results are presented as odds ratios (OR) with their corresponding 95% confidence intervals (CI). Finally, receiver operating characteristic (ROC) curve analysis was performed to evaluate the predictive value of the identified risk factors for *in-situ* hemorrhage. The optimal cut-off value was determined using the Youden index to maximize the combined sensitivity and specificity. To further assess the incremental predictive performance of multiple risk factors, the predicted probability derived from a binary logistic regression model incorporating both patient- and procedure-related predictors was subjected to ROC analysis. The area under the curve (AUC) with its 95% CI was calculated for both the single-factor and combined models.

## 3 Results

**FIGURE 1:**
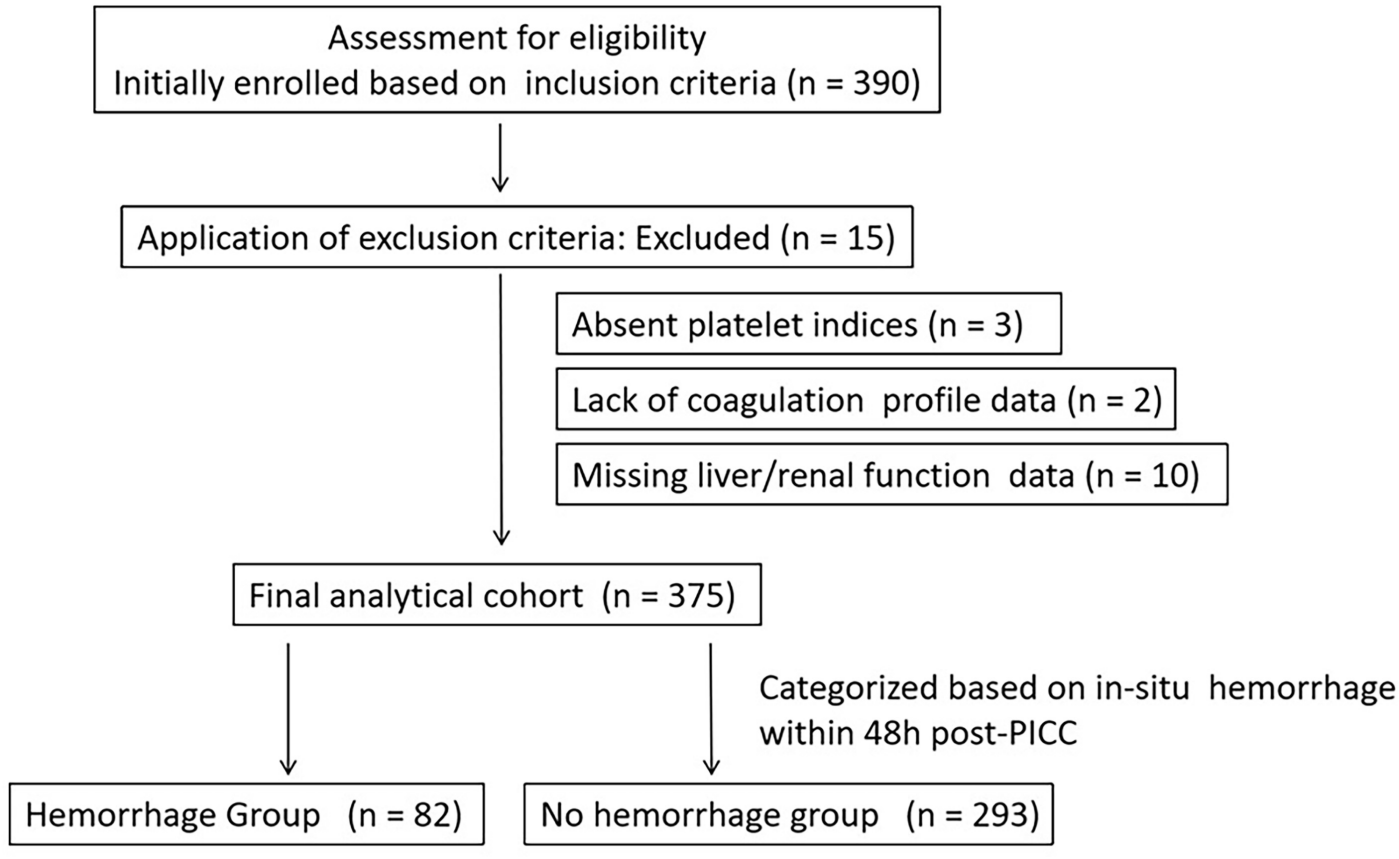
Patient enrollment and cohort formation process.

Based on the inclusion criteria, 390 patients were initially enrolled in this study. After applying the exclusion criteria, 15 patients were excluded, resulting in a final analytical cohort of 375 patients. Of these, 82 (21.9%) presented with *in-situ* hemorrhage within 48 h post-PICC, while 293 (78.1%) did not. Among the 15 excluded cases, the reasons were as follows: missing liver or renal function data (*n* = 10), absent platelet indices (*n* = 3), and lacking coagulation profile data (*n* = 2). Patient enrollment and cohort formation process was shown in Figure 1.

### 3.1 Comparison of patient-related data

The baseline characteristics of all patients, stratified by the presence or absence of *in-situ* hemorrhage within 48 h post-PICC, are presented in Table 1. Univariate comparisons revealed several significant differences between the bleeding and non-bleeding groups. Patients with hemorrhage were significantly older (median 72 vs. 66 years, *p* = 0.003) and included a lower proportion of males (36.6% vs. 50.9%, *p* = 0.025). Regarding primary diagnoses, acute ischemic stroke was more prevalent in the bleeding group (48.8% vs. 23.2%, *p* < 0.001), whereas tumors were more common in the non-bleeding group (36.6% vs. 67.9%, *p* < 0.001). Hypertension was also more frequent among patients with hemorrhage (54.9% vs. 41.3%, *p* = 0.033).

**TABLE 1:** Comparison of patient-related characteristics stratified by *in-situ* bleeding status within 48 h post-PICC.

Parameters	All (*n* = 375)	With *in-situ* bleeding (*n* = 82)	Without *in-situ* bleeding (*n* = 293)	*P*-value
Demographic factors				
Age, year	67 (57–75)	72 (59–82)	66 (56–73)	0.003
Male, *n* (%)	179 (47.7)	30 (36.6)	149 (50.9)	0.025
Disease status				
Primary diagnosis, *n* (%)				
Acute ischemic stroke	108 (28.8)	40 (48.8)	68 (23.2)	<0.001
Intracranial infection	28 (7.5)	8 (9.8)	20 (6.8)	0.351
Tumors	229 (61.1)	30 (36.6)	199 (67.9)	<0.001
Others	10 (2.7)	4 (4.9)	6 (2.0)	0.235
Comorbidities, *n* (%)				
Coronary heart disease	57 (15.2)	17 (20.7)	40 (13.7)	0.120
Diabetes mellitus	85 (22.7)	21 (25.6)	64 (21.8)	0.460
Hypertension	166 (44.3)	45 (54.9)	121 (41.3)	0.033
Hyperlipidemia (current)	16 (4.3)	1 (1.2)	15 (5.1)	0.212
Medication history				
Anticoagulant use, *n* (%)	90 (24.0)	26 (31.7)	64 (21.8)	0.064
Antiplatelet use, *n* (%)	89 (23.7)	29 (35.4)	60 (20.5)	0.005
SAPT	74 (19.7)	24 (29.3)	50 (17.1)	0.014
DAPT	15 (4.0)	5 (6.1)	10 (3.4)	0.273
Laboratory parameters				
Liver function				
ALT, U/L	17.4 (12.1–27.6)	16.6 (11.8–30.5)	17.4 (12.2–27.2)	0.968
AST, U/L	22.2 (16.9–31.0)	22.8 (16.7–37.9)	21.8 (16.9–30.2)	0.196
Albumin, g/L	37.9 (34.5–41.3)	38.0 (32.8–41.7)	37.9 (34.7–41.3)	0.854
Renal function				
Creatinine, μmol/L	65.0 (52.0–79.0)	63.5 (48.0–79.8)	65.0 (54.0–79.5)	0.309
Urea nitrogen, mmol/L	5.3 (4.2–7.0)	5.0 (4.2–6.8)	5.4 (4.2–7.1)	0.337
Coagulation profile				
PT, s	11.7 (11.0–12.9)	11.8 (11.1–13.1)	11.6 (11.0–12.8)	0.700
PTA, %	93 (86–103)	94 (86–104)	93 (85–103)	0.679
APTT, s	29.6 (27.6–31.5)	29.8 (28.1–32.7)	29.5 (27.4–31.3)	0.086
TT, s	14.0 (12.9–14.9)	14.2 (13.4–15.7)	13.8 (12.8–14.8)	0.898
Fibrinogen, g/L	3.62 (2.97–4.47)	3.25 (2.75–4.08)	3.75 (3.07–4.59)	0.001
D-dimer, mg/L	0.290 (0.165–0.590)	0.289 (0.140–0.607)	0.293 (0.175–0.596)	0.476
Platelet parameters				
Platelet count, ×10^9^/L	226 (177–277)	210 (167–267)	228 (181–282)	0.074
PCT, %	0.22 (0.17–0.27)	0.21 (0.17–0.27)	0.22 (0.17–0.27)	0.238
MPV, fL	9.7 (9.2–10.4)	9.9 (9.2–10.6)	9.7 (9.2–10.4)	0.178
PDW, %	11.5 (9.8–15.9)	12.7 (10.3–16.1)	11.2 (9.7–15.6)	0.006
P-LCR, %	23.9 (18.9–28.1)	24.9 (19.0–30.8)	22.4 (18.9–27.7)	0.069

Data are presented as median (interquartile range, IQR) for continuous variables and *n* (%) for categorical variables. *P*-values are derived from comparisons between the “With *in-situ* bleeding” group (*n* = 82) and the “Without *in-situ* bleeding” group (*n* = 293), using the Mann-Whitney U test for continuous variables and the Chi-square or Fisher’s exact test for categorical variables, as appropriate. A two-sided *p*-value < 0.05 was considered statistically significant. SAPT, single antiplatelet therapy; DAPT, dual antiplatelet therapy; ALT, alanine aminotransferase; AST, aspartate aminotransferase; PT, prothrombin time; PTA, prothrombin activity; APTT, activated partial thromboplastin time; TT, thrombin time; PCT, plateletcrit; MPV, mean platelet volume; PDW, platelet distribution width; P-LCR, platelet-large cell ratio.

Pharmacologically, the use of antiplatelet agents was significantly associated with bleeding (35.4% vs. 20.5%, *p* = 0.005), primarily driven by single antiplatelet therapy (29.3% vs. 17.1%, *p* = 0.014). The use of anticoagulants showed a trend toward significance (31.7% vs. 21.8%, *p* = 0.064).

Key laboratory distinctions were observed in the coagulation profile and platelet parameters. Patients with hemorrhage had significantly lower fibrinogen levels (median 3.25 g/L vs. 3.75 g/L, *p* = 0.001), and higher PDW (12.7% vs. 11.2%, *p* = 0.006). Additionally, platelet count (*p* = 0.074), APTT (*p* = 0.086), and P-LCR (*p* = 0.069) showed borderline non-significant trends.

### 3.2 Comparison of procedure-related data

Procedure-related factors are summarized in Table 2. A significant association was observed between the number of puncture attempts and the occurrence of *in-situ* hemorrhage (*p* < 0.001). Patients who experienced bleeding required a higher number of puncture attempts (mean 1.34) compared to those without bleeding (mean 1.10), with 20.7% of patients in the bleeding group requiring two attempts versus only 4.8% in the non-bleeding group. Furthermore, catheter dislodgement was more frequent in the hemorrhage group (11.0% vs. 3.4%, *p* = 0.010). The occurrence of difficult catheter advancement was also significantly associated with bleeding (17.1% vs. 4.4%, *p* < 0.001). In contrast, the choice of punctured vein (*p* = 0.272), daily infusion frequency (*p* = 0.535), and limb muscle tone (*p* = 0.336) did not differ significantly between the two groups. No statistically significant difference was observed in *in-situ* hemorrhage rates among three operators.

**TABLE 2:** Comparison of procedure-related characteristics stratified by *in-situ* bleeding status within 48 h post-PICC.

Parameters	All (*n* = 375)	With *in-situ* bleeding (*n* = 82)	Without *in-situ* bleeding (*n* = 293)	*P*-value
Punctured vein				0.272
Basilic vein, *n* (%)	334 (89.1)	71 (86.6)	263 (89.8)	
Cephalic vein, *n* (%)	20 (5.3)	3 (3.6)	17 (5.8)	
Median cubital vein, *n* (%)	11 (2.9)	4 (4.9)	7 (2.4)	
Brachial vein, *n* (%)	10 (2.7)	4 (4.9)	6 (2.0)	
Number of puncture attempts				<0.001
1 Attempt, *n* (%)	333 (88.8)	61 (74.4)	272 (92.8)	
2 Attempts, *n* (%)	31 (8.3)	17 (20.7)	14 (4.8)	
3 Attempts, *n* (%)	8 (2.1)	3 (3.7)	5 (1.7)	
≥4 Attempts, *n* (%)	3 (0.8)	1 (1.2)	2 (0.7)	
Infusion frequency				0.535
Once daily, *n* (%)	11 (2.9)	2 (2.4)	9 (3.1)	
Twice daily, *n* (%)	116 (30.9)	29 (35.4)	87 (29.7)	
Three times daily, *n* (%)	193 (51.5)	39 (47.6)	154 (52.6)	
≥Four times daily, *n* (%)	55 (14.7)	12 (14.6)	43 (14.7)	
Limb muscle tone				0.336
Normal, *n* (%)	328 (87.5)	69 (84.1)	259 (88.4)	
Decreased, *n* (%)	31 (8.3)	10 (12.2)	21 (7.2)	
Increased, *n* (%)	16 (4.3)	3 (3.7)	13 (4.4)	
Catheter dislodgement				0.010
With, *n* (%)	19 (5.1)	9 (11.0)	10 (3.4)	
Without, *n* (%)	356 (94.9)	73 (89.0)	283 (96.6)	
Catheter advancement difficulty				<0.001
With, *n* (%)	27 (7.2)	14 (17.1)	13 (4.4)	
Without, *n* (%)	348 (92.8)	68 (82.9)	280 (95.6)	
Bleeding rates by operator				0.794
Operator 1, *n* (%)	121 (32.3)	29 (24.0)	92 (76.0)	
Operator 2, *n* (%)	125 (33.3)	26 (20.8)	99 (79.2)	
Operator 3, *n* (%)	129 (34.4)	27 (20.9)	102 (79.1)	

*P*-values are derived from comparisons between the “With *in-situ* bleeding” group (*n* = 82) and the “Without *in-situ* bleeding” group (*n* = 293), using the Chi-square or Fisher’s exact test for categorical variables, as appropriate. A two-sided *p*-value < 0.05 was considered statistically significant. PICC, peripherally inserted central catheter.

### 3.3 Identification of independent predictors

Univariate binary logistic regression was first performed to identify potential patient-related risk and protective factors for *in-situ* hemorrhage within 48 h post-PICC. Candidate variables were selected based on significant between-group differences (*p* < 0.05), including age, sex, hypertension, primary diagnosis (acute ischemic stroke and tumors), use of antiplatelet agents, fibrinogen, and platelet distribution width. Given their borderline significance (*p* < 0.1) and established clinical relevance, anticoagulant use and platelet count were also included in the univariate analysis. The results confirmed that age, sex, acute ischemic stroke, tumors, hypertension, antiplatelet use, and fibrinogen level were significantly associated with *in-situ* hemorrhage.

These seven patient-related variables were subsequently entered into a multivariate binary logistic regression model (Model 1) to identify independent predictors, and two factors from patient-related data were identified as independent predictors of *in-situ* hemorrhage within 48 h post-PICC. A diagnosis of tumor was associated with a significantly lower risk of bleeding (OR = 0.331, 95% CI: 0.142–0.768, *p* = 0.010). Conversely, a lower level of fibrinogen was independently associated with an increased risk of hemorrhage (OR = 0.762 per 1 g/L increase, 95% CI: 0.583–0.995, *p* = 0.046). Other variables, including the use of antiplatelet agents, hypertension, a primary diagnosis of stroke, age and sex did not retain statistical significance in the multivariate model 1 (all *p* > 0.05). To control for potential confounding effects of procedural factors on the patient-related predictors, we constructed Model 2 by incorporating the three procedure-related variables that demonstrated significant between-group differences: catheter dislodgement, difficult catheter advancement, and number of puncture attempts. The results of this adjusted model remained consistent with those of Model 1, confirming a diagnosis of tumor and fibrinogen as the independent patient-related predictors of *in-situ* hemorrhage within 48 h post-PICC. The results of univariate and multivariate binary logistic regressions were shown in Table 3.

**TABLE 3:** Multiple analyses for independent patient-related risk/protective factors of *in-situ* bleeding within 48 h post-PICC.

Characteristic	Univariate analysis^a^		Multivariate analyses (model 1)^b^		Multivariate analyses (model 2)^c^	
	OR (95%CI)	*P*-value	OR (95%CI)	*P*-value	OR (95%CI)	*P*-value
Age	1.017 (1.000–1.033)	0.046	1.012 (0.994–1.030)	0.177	1.006 (0.988–1.025)	0.491
Proportion of males	1.794 (1.083–2.970)	0.023	1.499 (0.877–2.561)	0.138	1.507 (0.871–2.609)	0.143
Stroke (current)	3.151 (1.891–5.253)	<0.001	1.191 (0.452–3.139)	0.724	1.246 (0.465–3.340)	0.662
Tumor (current)	0.273 (0.163–0.455)	<0.001	0.331 (0.142–0.768)	0.010	0.349 (0.146–0.831)	0.017
Comorbid hypertension	1.729 (1.056–2.831)	0.030	1.132 (0.624–2.052)	0.684	1.101 (0.597–2.030)	0.757
Antiplatelet use	2.125 (1.245–3.625)	0.006	0.773 (0.369–1.617)	0.494	0.785 (0.369–1.669)	0.529
Fibrinogen level	0.716 (0.563–0.912)	0.007	0.762 (0.583–0.995)	0.046	0.757 (0.575–0.995)	0.046

^a^In univariate binary logistic regression, candidate variables were selected based on significant or borderline significant between-group differences (*p* < 0.1), considering their established clinical relevance.

^b^The sample size for Model 1, which included seven patient-related variables derived from original univariate logistc regression analyses, adhered to the general rule of thumb requiring a minimum of 10 events per variable, given that 82 hemorrhage events were recorded.

^c^The Model 2 was adjusted with three procedure-related variables that demonstrated significant between-group differences: catheter dislodgement, difficult catheter advancement, and number of puncture attempts. PICC, peripherally inserted central catheter; OR, odds ratios; CI: confidence intervals.

To further assess procedure-related factors as independent predictors, a series of logistic regression models were fitted, with the results summarized in Table 4. First, univariate analyses were performed for variables showing significant between-group differences (*p* < 0.05), including catheter dislodgement, number of puncture attempts, and difficult catheter advancement, all of which were identified as potential risk factors. These three variables were then included in a multivariable logistic regression model (Model 1). The results indicated that catheter advancement difficulty remained a significant independent predictor (OR = 2.560, 95% CI: 1.010–6.485, *p* = 0.047). The number of puncture attempts showed a trend toward significance (OR = 1.661 per attempt, 95% CI: 0.978–2.821, *p* = 0.060), while catheter dislodgement was not independently associated with bleeding risk (OR = 2.006, 95% CI: 0.699–5.752, *p* = 0.195). In Model 2, the analysis was further adjusted for seven patient-related confounders (age, sex, hypertension, primary diagnosis of acute ischemic stroke/tumors, use of antiplatelet agents, and fibrinogen level). The results of this adjusted model were consistent with those from Model 1.

**TABLE 4:** Multiple analyses for independent procedure-related risk/protective factors of *in-situ* bleeding within 48 h post-PICC.

Characteristic	Univariate analysis^a^		Multivariate analyses (model 1)^b^		Multivariate analyses (model 2)^c^	
	OR (95%CI)	*P*-value	OR (95%CI)	*P*-value	OR (95%CI)	*P*-value
Puncture attempts	2.218 (1.392–3.533)	0.001	1.661 (0.978–2.821)	0.060	1.326 (0.762–2.307)	0.318
Advancement difficulty	4.434 (1.992–9.870)	<0.001	2.560 (1.010–6.485)	0.047	3.211 (1.211–8.524)	0.019
Catheter dislodgement	3.489 (1.368–8.901)	0.009	2.006 (0.699–5.752)	0.195	1.469 (0.493–4.374)	0.490

^a^Univariate binary logistic regression analyses were performed for each procedure-related variable.

^b^Model 1: Multivariable binary logistic regression included three procedure-related variables that showed significant differences in univariate analyses (*p* < 0.05).

^c^Model 2: Multivariable binary logistic regression additionally adjusted for seven patient-related confounders that demonstrated significant between-group differences: age, sex, hypertension, primary diagnosis (acute ischemic stroke and tumors), use of antiplatelet agents, and fibrinogen level. PICC, peripherally inserted central catheter; OR, odds ratios; CI: confidence intervals.

Variance inflation factors for all predictors were <2.0, ruling out multicollinearity. The Hosmer-Lemeshow test showed adequate fit for both models (*p* = 0.404 and 0.397, respectively). Bootstrap internal validation (1,000 resamples) confirmed model stability. Our ROC analysis showed that a fibrinogen cut-off of 3.33 g/L had poor predictive value for early bleeding (AUC 0.381, sensitivity 43.9% and specificity 65.5%). When combined with catheter advancement difficulty, the AUC improved to 0.619, indicating that a multifactorial model outperforms fibrinogen alone. However, the overall diagnostic accuracy remains low-to-moderate, suggesting that clinical risk assessment should integrate multiple patient- and procedure-related factors rather than relying on a single biomarker threshold.

### 3.4 Subgroup analyses

Stratified analyses were performed to examine whether the effects of the identified predictors varied across subgroups. As shown in Supplementary Table 1, in the non-tumor subgroup, fibrinogen remained a significant protective factor (OR = 0.515, 95% CI: 0.321–0.828, *p* = 0.006), whereas catheter advancement difficulty was not significant (OR = 0.576, *p* = 0.502). In the tumor subgroup, catheter advancement difficulty was paradoxically associated with a reduced bleeding risk (OR = 0.067, 95% CI: 0.010–0.459, *p* = 0.006), but this finding should be interpreted with caution due to the limited sample size.

When stratified by antiplatelet use (Supplementary Table 2), catheter advancement difficulty emerged as a remarkably strong risk factor in patients not receiving antiplatelet therapy (OR = 17.351, 95% CI: 3.185–94.530, *p* = 0.001), while no significant association was observed in the antiplatelet user subgroup. Fibrinogen did not reach statistical significance in either stratum.

## 4 Discussion

This single-center retrospective study identified several factors potentially associated with *in-situ* hemorrhage within 48 h post-PICC insertion, a common yet clinically consequential complication. Through multivariable analysis, we identified fibrinogen level and catheter advancement difficulty as independent risk factors, alongside an unanticipated observation regarding tumor diagnosis, which appeared to be associated with a lower bleeding risk. While the latter finding warrants cautious interpretation and requires confirmation in future studies, the overall results reinforce existing clinical knowledge and offer preliminary insights that may inform risk stratification and targeted preventive strategies.

The most significant patient-related predictor was a lower fibrinogen level. As a crucial substrate for clot formation, fibrinogen directly governs the structural integrity and stability of the hemostatic plug formed at the puncture site (14, 15). Our finding aligns with the physiological principle of coagulation and the results of previous researches (15), providing a potential laboratory-based marker for bleeding risk that is readily available in clinical practice. Notably, the use of antiplatelet agents, while significant in univariate analysis, did not retain independence in the multivariate model. This suggests that its effect might be secondary to or confounded by a more potent underlying coagulopathy, such as low fibrinogen levels often seen in critically ill or malnourished patients. The observed discrepancy, wherein antiplatelet therapy was significantly associated with bleeding in the univariate analysis but not retained as an independent risk factor in the multivariate model, can be attributed to several considerations. Firstly, the inherent limitations of our single-center retrospective design, including a constrained sample size, may have reduced the statistical power to detect an independent effect. More importantly, the mere prescription of oral antiplatelet agents does not uniformly equate to effective platelet inhibition. The efficacy of antiplatelet therapy is highly variable and influenced by a multitude of factors. A key element is genetic polymorphism; for instance, the role of gene polymorphism in aspirin resistance is approximately 14%–39% (16), while the prevalence of poor metabolizer status for clopidogrel is notably high, affecting 4%–30% of the general population (17), with even higher rates reported in Chinese cohorts (18). Furthermore, underlying conditions such as renal insufficiency, diabetes, systemic inflammation, and anemia have been significantly linked to high on-treatment platelet reactivity and antiplatelet drug resistance (19). Additionally, drug-drug interactions can profoundly alter the absorption and metabolic activation of these agents (20). Consequently, future investigations employing more rigorous, detailed clinical trial designs and larger, multi-center prospective cohorts are warranted to provide a more definitive analysis of this relationship. Given that these mechanisms were not directly assessed in our study, the interpretations offered above remain speculative and should be viewed as exploratory.

Intriguingly, a diagnosis of tumor emerged as a “protective” factor. This seemingly counterintuitive result warrants careful interpretation. We hypothesize that this association may be attributed to a pro-thrombotic state frequently observed in cancer patients, particularly those with advanced disease (21). This state, characterized by elevated levels of pro-coagulant factors and enhanced platelet activation, might paradoxically facilitate more effective hemostasis at the venous puncture site. Alternatively, it could reflect a “selection bias” where our cohort of cancer patients was predominantly receiving chemotherapy or nutritional support, and thus might have been in a more stable clinical condition with better-preserved overall physiological reserves compared to the acutely ill patients with stroke or infections who constituted a larger proportion of the bleeding group. Furthermore, we acknowledge that cancer patients represent a heterogeneous population with diverse hemostatic profiles, and the lack of stratification by cancer type, stage, or treatment modality in the present analysis may have obscured important subgroup differences. Consequently, these findings should be interpreted as hypothesis-generating rather than definitive, and further studies with detailed cancer phenotyping are warranted to clarify the underlying mechanisms.

Our investigation into procedural determinants of early post-insertion hemorrhage revealed two critical factors: catheter advancement difficulty as an independent risk factor and a strong trend associating multiple puncture attempts with increased bleeding. The elucidation of these mechanisms provides a tangible pathway for refining clinical practice and improving patient safety. The identification of catheter advancement difficulty as an independent predictor of bleeding underscores a significant, yet potentially underappreciated, aspect of PICC placement. The pathophysiological rationale for this association is likely multifactorial. Primarily, difficult advancement often entails repeated back-and-forth manipulation of the catheter within the vein. This mechanical action can cause substantial trauma to the venous endothelium and the surrounding perivascular tissues, effectively enlarging the percutaneous tract. A larger, more traumatized tract presents a greater surface area for bleeding and disrupts a more extensive network of small capillaries, challenging the body’s hemostatic mechanisms. Furthermore, endothelial injury triggers a local inflammatory and pro-coagulant response; however, in the acute phase, the physical disruption may overwhelm nascent clot formation, leading to overt oozing or hematoma formation. While the number of puncture attempts showed a strong trend rather than achieving universal statistical significance as an independent predictor across all models, its clinical relevance is undeniable. The positive association aligns with the fundamental principle of tissue injury: each unsuccessful puncture attempt inflicts discrete trauma. This results in the creation of multiple tissue tracts and puncture sites in the vessel wall, causing cumulative damage.

These exploratory subgroup findings suggest that the effect of procedural difficulty may be masked by antiplatelet-induced hemostatic impairment, and that the predictive value of fibrinogen may be subgroup-dependent. Specifically, in patients not receiving antiplatelet therapy, catheter advancement difficulty emerged as an exceptionally strong risk factor (OR = 17.35), supporting the hypothesis that mechanical vascular injury is a primary driver of bleeding in the absence of pharmacological hemostatic derangement. Conversely, among patients on antiplatelet agents, the absence of a significant association may reflect a “ceiling effect,” wherein drug-induced platelet dysfunction dominates the bleeding risk, thereby attenuating the additional contribution of procedural factors. Similarly, the protective effect of fibrinogen observed in non-tumor patients, but not in those with malignancy, may be explained by the complex and heterogeneous hemostatic alterations associated with cancer—including hypercoagulability, abnormal fibrinolysis, and thrombocytopenia induced by chemotherapy—which collectively may obscure the predictive value of a single coagulation parameter.

Several limitations of this study should be acknowledged. First, its single-center, retrospective design inherently carries risks of selection bias and unmeasured confounding; for instance, the significant difference in sex distribution between the two groups may reflect underlying selection bias in the enrolled population. Second, certain procedural and laboratory variables could not be quantitatively standardized or fully captured. Third, key indicators reflecting the efficacy of antiplatelet therapy—such as arachidonic acid (AA)- and adenosine diphosphate (ADP)-induced maximum platelet aggregation rates, results of genetic polymorphism testing, and detailed records of concomitant medications affecting antiplatelet drug absorption and metabolism—were not collected. Fourth, we acknowledge that our binary definition does not capture the full spectrum of bleeding severity; the absence of a rigorous classification or grading system for bleeding severity may have influenced the accuracy of risk factor interpretation, as cases with minor oozing were grouped together with those of more extensive hematoma formation. Moreover, given the current lack of universally accepted criteria for defining early *in-situ* hemorrhage following PICC insertion, our proposed definition requires further validation in larger, well-designed clinical studies. Therefore, we recommend that future studies consider employing a validated, graded bleeding scale—such as the Bleeding Academic Research Consortium (BARC) criteria—to enable more precise risk stratification and to better characterize the clinical significance of post-insertion hemorrhage. Fifth, while the definition of “catheter dislodgement” was based on clinical assessment, minor catheter migrations may have been underreported. Sixth, we did not systematically capture comprehensive nutritional status parameters (e.g., body mass index, prealbumin, and hemoglobin levels) or detailed lipid profiles (e.g., cholesterol levels, which have been associated with bleeding risk), nor did we fully account for conditions such as uremia that can impair platelet function. Finally, the generalizability of our findings—particularly the unanticipated protective effect associated with a tumor diagnosis—requires validation in larger, multi-center, prospective populations.

Building upon the findings and limitations of this study, several critical avenues for future research emerge. First, a large-scale, multi-center prospective cohort study is essential to validate the identified independent risk factors—particularly the unanticipated protective effect of a tumor diagnosis—and to enhance the generalizability of the results. Such a study should systematically incorporate key variables missing from the present analysis, including objective measures of platelet function (e.g., AA-/ADP-induced platelet aggregation), genetic testing for antiplatelet drug resistance (e.g., CYP2C19 genotyping for clopidogrel), detailed nutritional profiles, and a standardized bleeding severity scale. Furthermore, multi-center studies with larger and more diverse operator cohorts are warranted to systematically evaluate the relationship between operator experience and early bleeding risk, as our single-center design with three experienced operators limited the ability to detect operator-related variability. Second, the underlying mechanisms warrant deeper exploration; specifically, research into the potential “pro-thrombotic” state in cancer patients and its relationship with PICC-related bleeding risk could elucidate the observed “protective” association. Finally, the development and testing of targeted intervention strategies are urgently needed. Prospective trials should evaluate whether protocolized management of pre-procedural hypo-fibrinogenemia or the implementation of advanced catheter securement devices can effectively reduce the incidence of early hemorrhage, ultimately translating these findings into improved clinical practice and patient safety.

## 5 Conclusion

In conclusion, this study establishes lower fibrinogen levels and catheter advancement difficulty as independent risk factors for early PICC-related hemorrhage, while revealing a potential protective effect of tumor diagnosis possibly mediated by a hypercoagulable state. These findings underscore the clinical value of pre-procedural fibrinogen assessment and refinement of catheter placement protocols. Future multi-center studies should validate these relationships and explore targeted interventions to mitigate bleeding risk and improve PICC safety outcomes.

## Data Availability

The original contributions presented in this study are included in this article/Supplementary material, further inquiries can be directed to the corresponding authors.
