# Supplementary material for: Fibrinogen and catheter advancement difficulty: unveiling the key predictors of early in-situ bleeding after peripherally inserted central catheter insertion
Source: Front Med (Lausanne). 2026 Jul 15;13:1836789. doi: 10.3389/fmed.2026.1836789 (PMC13415919; doi:10.3389/fmed.2026.1836789)
Supplement: Supplementary file 1 [file Table_1.docx]

**Supplementary Table 1**. Subgroup analysis of risk factors for in-situ hemorrhage stratified by tumor status

| Characteristics | Without tumor | |  | With tumor | |
| --- | --- | --- | --- | --- | --- |
|  | OR (95%CI) | P value |  | OR (95%CI) | P value |
| Fibrinogen level | 0.515 (0.321-0.828) | 0.006 |  | 1.137 (0.807-1.602) | 0.464 |
| Advancement difficulty | 0.576 (0.115-2.890) | 0.502 |  | 0.067 (0.010-0.459) | 0.006 |

**Supplementary Table 2**. Subgroup analysis of risk factors for in-situ hemorrhage stratified by antiplatelet status

| Characteristics | Without antiplatelet | |  | With antiplatelet | |
| --- | --- | --- | --- | --- | --- |
|  | OR (95%CI) | P value |  | OR (95%CI) | P value |
| Fibrinogen level | 0.798 (0.581-1.095) | 0.162 |  | 0.638 (0.334-1.217) | 0.173 |
| Advancement difficulty | 17.351 (3.185-94.530) | 0.001 |  | 0.310 (0.037-2.609) | 0.281 |
